# Comparative Treatment Outcomes for Patients With Idiopathic Subglottic Stenosis

**DOI:** 10.1001/jamaoto.2019.3022

**Published:** 2019-10-31

**Authors:** Alexander Gelbard, Catherine Anderson, Lynne D. Berry, Milan R. Amin, Michael S. Benninger, Joel H. Blumin, Jonathan M. Bock, Paul C. Bryson, Paul F. Castellanos, Sheau-Chiann Chen, Matthew S. Clary, Seth M. Cohen, Brianna K. Crawley, Seth H. Dailey, James J. Daniero, Alessandro de Alarcon, Donald T. Donovan, Eric S. Edell, Dale C. Ekbom, Sara Fernandes-Taylor, Daniel S. Fink, Ramon A. Franco, C. Gaelyn Garrett, Elizabeth A. Guardiani, Alexander T. Hillel, Henry T. Hoffman, Norman D. Hogikyan, Rebecca J. Howell, Li-Ching Huang, Lena K. Hussain, Michael M. Johns, Jan L. Kasperbauer, Sid M. Khosla, Cheryl Kinnard, Robbi A. Kupfer, Alexander J. Langerman, Robert J. Lentz, Robert R. Lorenz, David G. Lott, Anne S. Lowery, Samir S. Makani, Fabien Maldonado, Kyle Mannion, Laura Matrka, Andrew J. McWhorter, Albert L. Merati, Matthew C. Mori, James L. Netterville, Karla O’Dell, Julina Ongkasuwan, Gregory N. Postma, Lindsay S. Reder, Sarah L. Rohde, Brent E. Richardson, Otis B. Rickman, Clark A. Rosen, Michael J. Rutter, Guri S. Sandhu, Joshua S. Schindler, G. Todd Schneider, Rupali N. Shah, Andrew G. Sikora, Robert J. Sinard, Marshall E. Smith, Libby J. Smith, Ahmed M. S. Soliman, Sigríður Sveinsdóttir, Douglas J. Van Daele, David Veivers, Sunil P. Verma, Paul M. Weinberger, Philip A. Weissbrod, Christopher T. Wootten, Yu Shyr, David O. Francis

**Affiliations:** 1 Department of Otolaryngology–Head and Neck Surgery, Vanderbilt University Medical Center, Nashville, Tennessee; 2 Vanderbilt Center for Quantitative Sciences, Vanderbilt-Ingram Cancer Center, Nashville, Tennessee; 3 New York University Voice Center, Department of Otolaryngology–Head and Neck Surgery, New York University School of Medicine, New York; 4 Department of Otolaryngology–Head and Neck Surgery, Head and Neck Institute, Cleveland Clinic, Cleveland, Ohio; 5 Division of Laryngology and Professional Voice, Department of Otolaryngology and Communication Sciences, Medical College of Wisconsin, Milwaukee; 6 Division of Otolaryngology, Department of Surgery, University of Alabama at Birmingham, Birmingham; 7 Department of Otolaryngology–Head and Neck Surgery, University of Colorado School of Medicine, Denver; 8 Duke Voice Care Center, Division of Otolaryngology–Head and Neck Surgery, Duke University Medical Center, Durham, North Carolina; 9 Department of Otolaryngology–Head & Neck Surgery, Loma Linda University Health, Loma Linda, California; 10 Department of Surgery, University of Wisconsin Hospitals and Clinics, Madison; 11 Department of Otolaryngology–Head and Neck Surgery, University of Virginia Health System, Charlottesville; 12 Department of Otolaryngology–Head and Neck Surgery, University of Cincinnati, Cincinnati, Ohio; 13 Department of Otolaryngology–Head and Neck Surgery, Baylor College of Medicine, Houston, Texas; 14 Division of Pulmonary and Critical Care Medicine, Mayo Clinic, Rochester, Minnesota; 15 Department of Otolaryngology, Mayo Clinic College of Medicine and Science, Rochester, Minnesota; 16 Department of Otolaryngology–Head and Neck Surgery, Massachusetts Eye and Ear Infirmary, Boston; 17 Department of Otorhinolaryngology–Head and Neck Surgery, University of Maryland School of Medicine, Baltimore; 18 Department of Otolaryngology–Head and Neck Surgery, Johns Hopkins Medical Institution, Baltimore, Maryland; 19 Department of Otolaryngology–Head and Neck Surgery, University of Iowa Hospitals and Clinics, Iowa City; 20 Department of Otolaryngology–Head and Neck Surgery, University of Michigan Medical Center, Ann Arbor; 21 Department of Otolaryngology–Head and Neck Surgery, University of Southern California, Los Angeles; 22 Division of Allergy, Pulmonary and Critical Care Medicine, Vanderbilt University Medical Center, Nashville, Tennessee; 23 Department of Otorhinolaryngology, Mayo Clinic Scottsdale, Scottsdale, Arizona; 24 Division of Pulmonary and Critical Care Medicine, University of California, San Diego, San Diego; 25 Department of Otolaryngology–Head & Neck Surgery, The Ohio State University Medical Center, Columbus; 26 Department of Otolaryngology, Louisiana State University Health Sciences Center-New Orleans, New Orleans; 27 Department of Otolaryngology–Head & Neck Surgery, University of Washington Medical Center, Seattle; 28 Department of Otolaryngology, New York Eye and Ear Infirmary of Mount Sinai, New York; 29 Department of Otolaryngology–Head and Neck Surgery, Medical College of Georgia at Augusta University, Augusta; 30 Bastian Voice Institute, Downers Grove, Illinois; 31 Department of Otolaryngology–Head and Neck Surgery, University of California, San Francisco, San Francisco; 32 National Centre for Airway Reconstruction, Charing Cross Hospital, Imperial College Healthcare National Health System Trust, London, United Kingdom; 33 Department of Otolaryngology–Head & Neck Surgery, Northwest Clinic for Voice and Swallowing, Oregon Health and Science University, Portland; 34 Department of Otolaryngology, University of Rochester, Rochester, New York; 35 Department of Otolaryngology–Head and Neck Surgery, University of North Carolina at Chapel Hill, Chapel Hill; 36 Division of Otolaryngology–Head & Neck Surgery, The University of Utah, Salt Lake City; 37 Department of Otolaryngology–Head and Neck Surgery, University of Pittsburgh, Pittsburgh, Pennsylvania; 38 Department of Otolaryngology–Head and Neck Surgery, Lewis Katz School of Medicine at Temple University, Philadelphia, Pennsylvania; 39 Landspítali University Hospital, Reykjavik, Iceland; 40 Department of Otolaryngology–Head and Neck Surgery, Royal North Shore Hospital, Sydney, Australia; 41 Department of Otolaryngology–Head and Neck Surgery, University of California, Irvine; 42 Departments of Otolaryngology, Molecular and Cellular Physiology, Feist-Weiller Cancer Center, Louisiana State University, Shreveport; 43 Division of Otolaryngology–Head & Neck Surgery, Department of Surgery, University of California, San Diego, San Diego

## Abstract

**Importance:**

Surgical treatment comparisons in rare diseases are difficult secondary to the geographic distribution of patients. Fortunately, emerging technologies offer promise to reduce these barriers for research.

**Objective:**

To prospectively compare the outcomes of the 3 most common surgical approaches for idiopathic subglottic stenosis (iSGS), a rare airway disease.

**Design, Setting, and Participants:**

In this international, prospective, 3-year multicenter cohort study, 810 patients with untreated, newly diagnosed, or previously treated iSGS were enrolled after undergoing a surgical procedure (endoscopic dilation [ED], endoscopic resection with adjuvant medical therapy [ERMT], or cricotracheal resection [CTR]). Patients were recruited from clinician practices in the North American Airway Collaborative and an online iSGS community on Facebook.

**Main Outcomes and Measures:**

The primary end point was days from initial surgical procedure to recurrent surgical procedure. Secondary end points included quality of life using the Clinical COPD (chronic obstructive pulmonary disease) Questionnaire (CCQ), Voice Handicap Index-10 (VHI-10), Eating Assessment Test-10 (EAT-10), the 12-Item Short-Form Version 2 (SF-12v2), and postoperative complications.

**Results:**

Of 810 patients in this cohort, 798 (98.5%) were female and 787 (97.2%) were white, with a median age of 50 years (interquartile range, 43-58 years). Index surgical procedures were ED (n = 603; 74.4%), ERMT (n = 121; 14.9%), and CTR (n = 86; 10.6%). Overall, 185 patients (22.8%) had a recurrent surgical procedure during the 3-year study, but recurrence differed by modality (CTR, 1 patient [1.2%]; ERMT, 15 [12.4%]; and ED, 169 [28.0%]). Weighted, propensity score–matched, Cox proportional hazards regression models showed ED was inferior to ERMT (hazard ratio [HR], 3.16; 95% CI, 1.8-5.5). Among successfully treated patients without recurrence, those treated with CTR had the best CCQ (0.75 points) and SF-12v2 (54 points) scores and worst VHI-10 score (13 points) 360 days after enrollment as well as the greatest perioperative risk.

**Conclusions and Relevance:**

In this cohort study of 810 patients with iSGS, endoscopic dilation, the most popular surgical approach for iSGS, was associated with a higher recurrence rate compared with other procedures. Cricotracheal resection offered the most durable results but showed the greatest perioperative risk and the worst long-term voice outcomes. Endoscopic resection with medical therapy was associated with better disease control compared with ED and had minimal association with vocal function. These results may be used to inform individual patient treatment decision-making.

## Key Points

**Question:**

What are the outcomes of the 3 most common surgical approaches for idiopathic subglottic stenosis (iSGS)?

**Findings:**

In this cohort study of 810 patients with iSGS who underwent 1 of the 3 most common surgical treatments, 23% of patients underwent a recurrent surgical procedure during the 3-year study period, but recurrence differed by modality (cricotracheal resection, 1%; endoscopic resection with adjuvant medical therapy, 12%; and endoscopic dilation, 28%). Among successfully treated patients, those who underwent cricotracheal resection reported the highest quality of life but the greatest perioperative risk and worst long-term voice outcomes.

**Meaning:**

These results show the feasibility of integrating an engaged rare disease community with a network of surgeons to facilitate rapid and nuanced treatment comparisons; findings may help inform treatment decision-making in iSGS.

## Teaser

This cohort study of 810 patients with idiopathic subglottic stenosis compares the outcomes of the 3 most common surgical treatment procedures: endoscopic dilation, endoscopic resection with adjuvant medication, or cricotracheal resection.

## Introduction

The paradox of rare diseases is that any single diagnosis affects a small number of individuals, but 6% to 8% of the world population is afflicted.^1^ This fact renders rare diseases both difficult to study and a significant public health concern. Even basic epidemiologic studies of rare diseases are challenging given the heterogeneous, progressive clinical course of the rare disease and the geographic dispersion of patients.^2^ Geographic distribution requires an extensive recruitment and monitoring infrastructure, dramatically increasing the cost and time required for participant accrual. These barriers, coupled with small markets, reduce incentives for the pharmaceutical industry and funders to support rare disease research,^3^ constrain evidence, and result in clinical practice variation and inconsistent patient outcomes.

Fortunately, emerging technologies offer promise to reduce the barriers to research about rare disease. Patients have begun to cluster in online communities to provide mutual support and information about their conditions.^4^ These communities have become rich sources of knowledge about the lived experience of patients with rare disease.^5^ Researchers are increasingly interested in leveraging online communities to rapidly accrue sample sizes required to generate evidence and improve treatments. Paralleling this trend are advances in personal mobile computing to generate biomedically relevant data streams, including self-monitoring of disease symptoms and vital signs. Patient-generated health data may hold immense promise to improve treatment outcomes by providing clinicians and researchers a view of disease progression at home.^6^

Idiopathic subglottic stenosis (iSGS) is a rare,^7^ recurrent, and devastating fibroinflammatory disease that leads to upper airway narrowing and severe dyspnea among adult white women.^8^ Because of high recurrence rates, more than half of patients with iSGS require repeated surgical procedures within 12 months of their initial diagnosis.^9^ Three treatments for iSGS predominate,^7,10,11^ and evidence has shown variability in outcomes.^8,11^ This variability has complicated patient decision-making as patients try to balance survival, symptoms, and quality-of-life considerations.^12^ We designed a prospective multicenter observational study comparing the effectiveness of the 3 most common treatments for iSGS using outcomes that matter most to patients: time to disease recurrence and treatment quality-of-life trade-offs. Harnessing emerging technologies, we directly recruited people with iSGS from an online community on Facebook in addition to traditional physician-led recruitment efforts. We used a novel approach to monitor treatment response that included clinical data from electronic health records, longitudinal physiologic data recorded in a smartphone app, and patient-reported outcome measures. By coupling patient-generated health data from the digital platform with clinical data from electronic health records, the burden of studying rare disease longitudinally across multiple sites was reduced. This approach facilitated rapid establishment of a study cohort and nuanced treatment comparisons.

## Methods

### Participants

Adult patients (≥18 years) with untreated, newly diagnosed, or previously treated iSGS meeting established diagnostic criteria were enrollment candidates.^8^ Age, sex, and race/ethnicity were collected based on recorded electronic medical records and confirmed with the patients. Recruitment of patients took place from June 1, 2015, to June 1, 2017. Patients with obstructing subglottic stenosis not attributable to the 2 most common etiologies (granulomatosis with polyangiitis and intubation-related airway trauma) were included.^8^ Patients were excluded if their index operative date was not confirmed or they failed to complete required baseline surveys. The study was approved by the Vanderbilt University Medical Center institutional review board, Nashville, Tennessee, and written informed consent was obtained electronically from each participant.

### Setting

Patients were recruited using both traditional and novel methods with a goal of enrolling 300 participants. The traditional method involved patient identification and recruitment by clinicians participating in the North American Airway Collaborative (NoAAC) network. The NoAAC consists of 30 participating tertiary care centers across all regions of the United States as well as international sites in Australia, France, Iceland, Norway, and the United Kingdom. All NoAAC sites are referral centers for iSGS and thus have significant experience treating this rare disease.^7,8,9,13,14,15^

The novel recruitment method involved direct patient enrollment from a community of patients with iSGS on Facebook. The online community “Living with Idiopathic Subglottic Stenosis” currently has 3030 members (2636 are patients with iSGS); 42% of members visit the site daily, and 97% visit at least monthly. The online community has robust leadership, and the founder and chief moderator is a person with iSGS. Her stewardship engenders a positive culture of balanced information sharing.^5^ The NoAAC engaged the online community leader to solicit her formal involvement in study planning. In addition to aligning patient and clinician goals, this integration allowed information (both initial recruiting efforts and continuous project updates) to flow from the leader to the online community. Weekly conversations between the leader and principal investigators kept community members updated on recruitment. Annual in-person study meetings (with all online community members invited to attend) maintained participant engagement. These features allowed study information to be rapidly disseminated. Interested online community members with iSGS directly enrolled by contacting the study coordinator, who obtained permission for medical records release and who completed consent for the patients electronically. Patient information collected from each recruitment method was screened to confirm inclusion criteria and to allow data entry into the secure electronic data repository.

### Study Protocol

#### Baseline

The study followed the Strengthening the Reporting of Observational Studies in Epidemiology (STROBE) reporting guideline and a prespecified protocol (eFigure in the Supplement).^16^ At enrollment, patients completed baseline surveys and a series of patient-reported outcome measures evaluating constructs affected by the disease and its treatment. In addition, disease-specific data, a mucosal atopy index, and comorbidity scores^17,18,19,20,21^ were abstracted from medical records.

#### Index Intervention

Each NoAAC center submitted its standard-of-care treatment algorithm before beginning enrollment. Symptomatic patients underwent standard-of-care treatment at their respective medical centers. The index, or most proximate, surgical procedure (if the last treatment predated study inception) was defined as time zero (T_0_).

Primary treatments were endoscopic dilation (ED), endoscopic resection with adjuvant medical therapy (ERMT), and open cricotracheal resection (CTR). All treatments are described in detail elsewhere.^16^ In brief, ED involves using a balloon or rigid bougie to expand the stenotic segment; ERMT uses a carbon dioxide laser to endoscopically resect quadrants of the stenotic airway followed by long-term adjuvant medication (eg, inhaled corticosteroid, a proton pump inhibitor, and trimethoprim/sulfamethoxazole); and CTR is an open surgical procedure that involves en bloc removal of the stenotic airway followed by anastomosis of the proximal and distal tracheal segments. The study nurse coordinator (C.K.) reviewed the specifics of the index surgical procedures to confirm the type of intervention and to ensure adherence to their standard of care.

#### Longitudinal Surveillance

Patients completed the electronic health status every 3 months after enrollment. The electronic data capture system generated a scheduled, automated query that solicited data on adjuvant medication use (eg, inhaled corticosteroids); whether patients had undergone any interventions to treat their iSGS (surgical or clinic-based) and, if so, whether they had experienced specific treatment-associated complications; and patient-reported outcome measures to track symptoms and quality of life (every 6 months). Reports of treatment or complications triggered notification of the study nurse coordinator, who investigated, verified, and documented these events, including the date of any recurrent surgical procedure. Patients were given the ability to self-monitor their disease status by daily recording of peak expiratory flow rate (PEFR) using an inexpensive portable handheld device and a free smartphone app designed by the authors.^22^ The PEFR was measured in liters per second during a single expiratory cycle and was reported as percentage of matched normative data (%PEFR).^23,24^

### Outcomes

The primary end point was need for and time to recurrent surgical procedure, defined as days from T_0_ to recurrent surgical procedure (T_R_). This end point was derived from semistructured interviews among patients with iSGS and participating clinicians, who agreed that it was the most appropriate surrogate for recurrence.^25^ Secondary end points included quality-of-life trade-offs and complications across treatment modalities.

### Statistical Analysis

#### Primary Outcome

Data was analyzed between July 1 and September 30, 2018. The Kaplan-Meier method assessed time to recurrent surgical procedure for the 3 primary treatments, and hazard ratios (HRs) and 95% CIs were calculated using weighted Cox proportional hazards regression models. Censoring occurred with a recurrent surgical procedure or a patient death. Propensity score matching and multiple imputation–adjusted HRs were used for known confounders and missing data (eMethods in the Supplement). Effect sizes (ESs) and 95% CIs were reported for all comparisons as indicated.

#### Indication for Recurrent Surgical Procedure

The observational study design precluded prespecifying indications for repeat surgical procedure. In general, the primary indication for repeat surgical procedure was worsened breathing; however, the threshold for repeat surgical procedure may differ across centers. Physiologic (%PEFR) and patient-reported Clinical COPD (chronic obstructive pulmonary disease) Questionnaire (CCQ) metrics were used to assess whether this threshold systematically differed by treatment modality. Loess curves of mixed-effects model–fitted values for %PEFR assessed the stability of respiratory function for nonrecurring patients.

## Results

### Recruitment and Study Population

**Figure 1.**
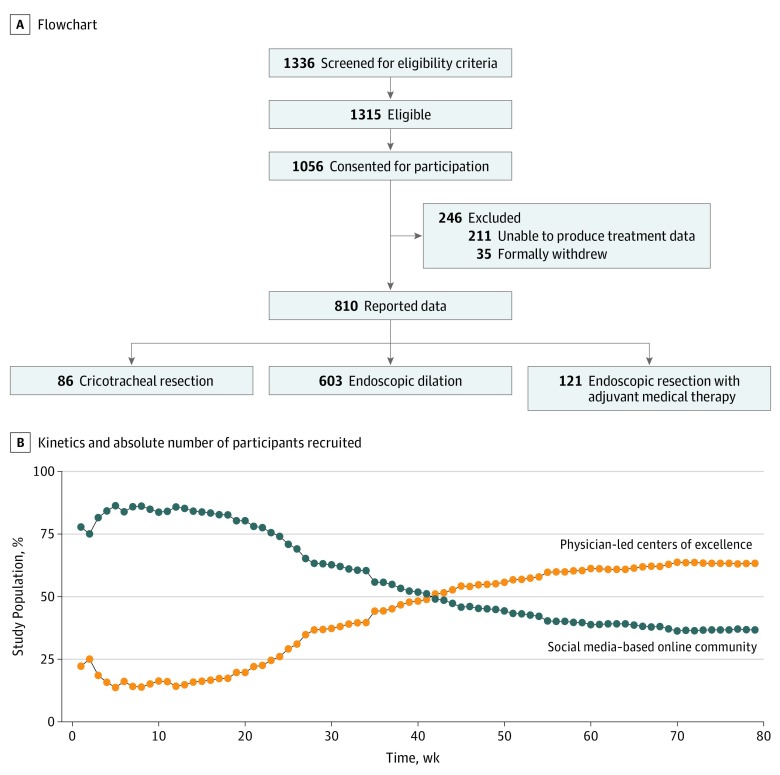
Flowchart and Absolute Number of Participants Recruited

Of the 1056 patients consented, 383 patients (36.3%) were recruited via social media, and 673 (63.7%) were recruited via physician referral (Figure 1A). Patient recruitment via social media outpaced that via physician referral during the first 47 weeks of the 3-year study (Figure 1B). Patients recruited via social media were slightly younger (mean [SD] age of recruits: social media, 47 [10] years vs physician referral, 53 [12] years) but otherwise lacked differences in sociodemographic or baseline survey completion rates. Given their earlier enrollment in the study, patients recruited via social media had a longer mean (SD) follow-up (1.47 [0.88] years vs 1.27 [0.79] years). Overall, 35 patients (3.3%) withdrew from the study, and 211 (20.6%) consented to participate but were unable to produce treatment data associated with their disease and the index procedure. Exclusions were balanced between Facebook recruits (71 of 368; 19.3%) and physician referrals (140 of 653; 21.4%).

In all, 810 patients meeting inclusion criteria enrolled, of whom 798 (98.5%) were female, 787 (97.2%) were white, and 64.8% (487 of 752 reporting) held college or advanced degrees, with a median age of 50 years (interquartile range [IQR], 43-58 years). Index operations were ED (n = 603; 74.4%), ERMT (n = 121; 14.9%), and CTR (n = 86; 10.6%) (Figure 1A). Anatomically, patients with iSGS presented with a median (IQR) subglottic narrowing of 11 mm (8-17 mm) caudal to the vocal folds, median (IQR) craniocaudal length of 15 mm (10-20 mm), and median (IQR) airway obstruction at T_0_ of 60% (50%-75%).

Patient sociodemographic and clinical characteristics were similar across treatment modalities (Table). Patients who underwent ERMT were predominantly white (121 of 121; 100%), older (median, 56 years; range, 48-63 years), and had greater median percentage of luminal obstruction at T_0_ (75%; range, 64%-80%). Patients who underwent ED showed the shortest median segment of subglottal narrowing (12 mm; range, 10-17 mm). Stenosis observed in this group was the farthest from the vocal folds (15 mm; range, 10-20 mm). Patients who underwent ERMT experienced the longest median duration of disease (8.6 years; IQR, 3.4-13.0 years), and patients who underwent CTR had the most surgical procedures (n = 5) performed before open reconstruction at T_0_ (range, 3-7 surgical procedures).

**Table.** Characteristics of Patients With Idiopathic Subglottic Stenosis

Characteristic	ED (n = 603)	ERMT (n = 121)	CTR (n = 86)	Total (N = 810)	Effect Size^a^	Test Type^b^
Age at index procedure, median (IQR), y	49 (42-57)	56 (48-63)	48 (39-55)	50 (43-58)	0.036 (0.015-0.063)	1
Female, No. (%)	593 (98.3)	121 (100)	84 (97.5)	798 (98.5)	0.032 (0-0.089)	2
Married, No. (%)^c^	426 (76.1)	86 (76.1)	70 (86.4)	582 (77.2)	0.076 (0-0.141)	2
White race, No. (%)	580 (95.9)	121 (100)	86 (100)	787 (97.2)	0.105 (0.017-0.171)	2
White (non-Hispanic or Latino) ethnicity, No. (%)^c^	503 (97.4)	87 (100)	60 (93.8)	650 (97.5)	0.089 (0-0.159)	2
Educational level, No. (%)^c^						
Graduate school	153 (27.4)	22 (19.5)	14 (17.3)	189 (25.1)	0.092 (0-0.127)	2
College graduate	218 (39.0)	42 (37.2)	38 (46.9)	298 (39.6)		
Some college	118 (21.0)	34 (30.1)	23 (28.4)	175 (23.3)		
High school or less	70 (12.5)	15 (13.3)	5 (6.2)	90 (12.0)		
Stenosis, median (IQR), %	50 (40-70)	75 (64-80)	60 (60-70)	60 (50-75)	0.06 (0.034-0.088)	1
Stenosis length, median (IQR), mm	12 (10-17)	15 (10-20)	17 (15-20)	15 (10-20)	0.02 (0.005-0.042)	1
Distance below glottis, median (IQR), mm	15 (10-20)	10 (5-15)	10 (5-15)	11 (8-17)	0.018 (0.005-0.04)	1
Disease duration, median (IQR), y	5.5 (2.5-9.9)	8.6 (3.4-13.0)	6.1 (3.9-10.2)	5.8 (2.6-10.7)	0.014 (0.002-0.034)	1
Surgical procedure, No. (range)	3 (2-7)	3 (2-6)	5 (3-7)	3 (2-7)	0.015 (0.004-0.031)	1
Charlson Comorbidity Index, median (IQR)	0	0	0	0	0.007 (0-0.018)	1
Gastroesophageal reflux disease, No. (%)^c^	206 (37.1)	43 (38.4)	31 (38.8)	280 (37.5)	0.013 (0-0.05)	2
Premenopausal, No. (%)^c^	204 (72.6)	20 (64.5)	25 (71.4)	249 (72.0)	0.051 (0-0.138)	2
Hormone replacement therapy, No. (%)^c^	5 (1.8)	1 (3.2)	0	6 (1.7)	0.054 (0-0.143)	2
Full-term pregnancy, No. (%)						
0	139 (25.3)	12 (10.9)	13 (16.5)	164 (22.2)	0.123 (0.041-0.157)	2
1	81 (14.7)	10 (9.1)	7 (8.9)	98 (13.3)		
2	203 (36.9)	51 (46.4)	35 (43.8)	289 (39.1)		
3	88 (16.0)	22 (20.0)	16 (20.0)	126 (17.1)		
>3	39 (7.1)	15 (13.6)	8 (10.0)	62 (8.4)		
Years of follow-up, median (IQR), y	1.3 (0.4-2.2)	1.5 (0.2-3.6)	4.3 (1.7-6.1)	1.4 (0.4-2.5)	0.011 (0.002-0.024)	1

Abbreviations: CTR, cricotracheal resection; ED, endoscopic dilation; ERMT, endoscopic resection with adjuvant medical therapy; IQR, interquartile range.

^a^ Epsilon-squared for Kruskal-Wallis test and Cramer V for Pearson χ^2^ test. The 95% CIs for epsilon-squared values are estimated by adjusted bootstrap percentile (bias-corrected and accelerated) interval with 1000 replications; 95% CIs for Cramer V values are estimated by noncentral χ^2^.

^b^ Test type: 1, Kruskal-Wallis test; 2, Pearson χ^2^ test.

^c^ Not reported for all participants.

### Time to Recurrence (Primary End Point)

**Figure 2.**
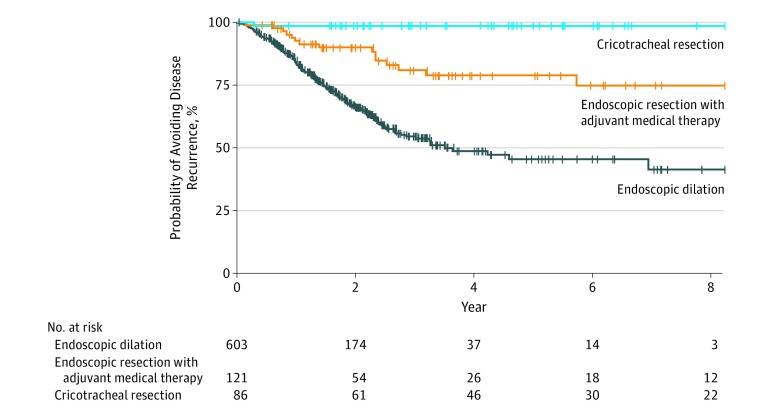
Kaplan-Meier Analysis of Disease Recurrence Among the 3 Treatment Groups

Median (IQR) follow-up after T_0_ was similar between patients who underwent ED (1.3 [0.4-2.2] years) vs those who underwent ERMT (1.5 [0.2-3.6] years) and was significantly longer for patients who underwent CTR (4.3 [1.7-6.1] years) (Table). Overall, 185 patients (22.8%) had a recurrent surgical procedure during the 3-year study period, but the rate differed across modalities. Open cricotracheal resection had a 1.2% recurrence rate (1 of 86 patients), followed by 12.4% (15 of 121) for ERMT and 28.0% (169 of 603) for ED. Kaplan-Meier analysis showed the need for and the time to recurrent surgical procedure between treatment modalities (Figure 2). With 1 recurrence within the CTR group, only ED and ERMT were comparable (ERMT vs ED: HR, 3.03; 95% CI, 1.78-5.17). Propensity score matching was used to minimize bias from nonrandomized treatment assignment. Weighted Cox proportional hazards regression models (eTable 1 in the Supplement) showed that ED was inferior compared with ERMT (HR, 2.77; 95% CI, 1.4-5.5), and this association persisted in propensity score–matching models accounting for missing data (ED vs ERMT: HR, 3.16; 95% CI, 1.82-5.51) (eTable 2 in the Supplement).

### Indication for Recurrent Operation

**Figure 3.**
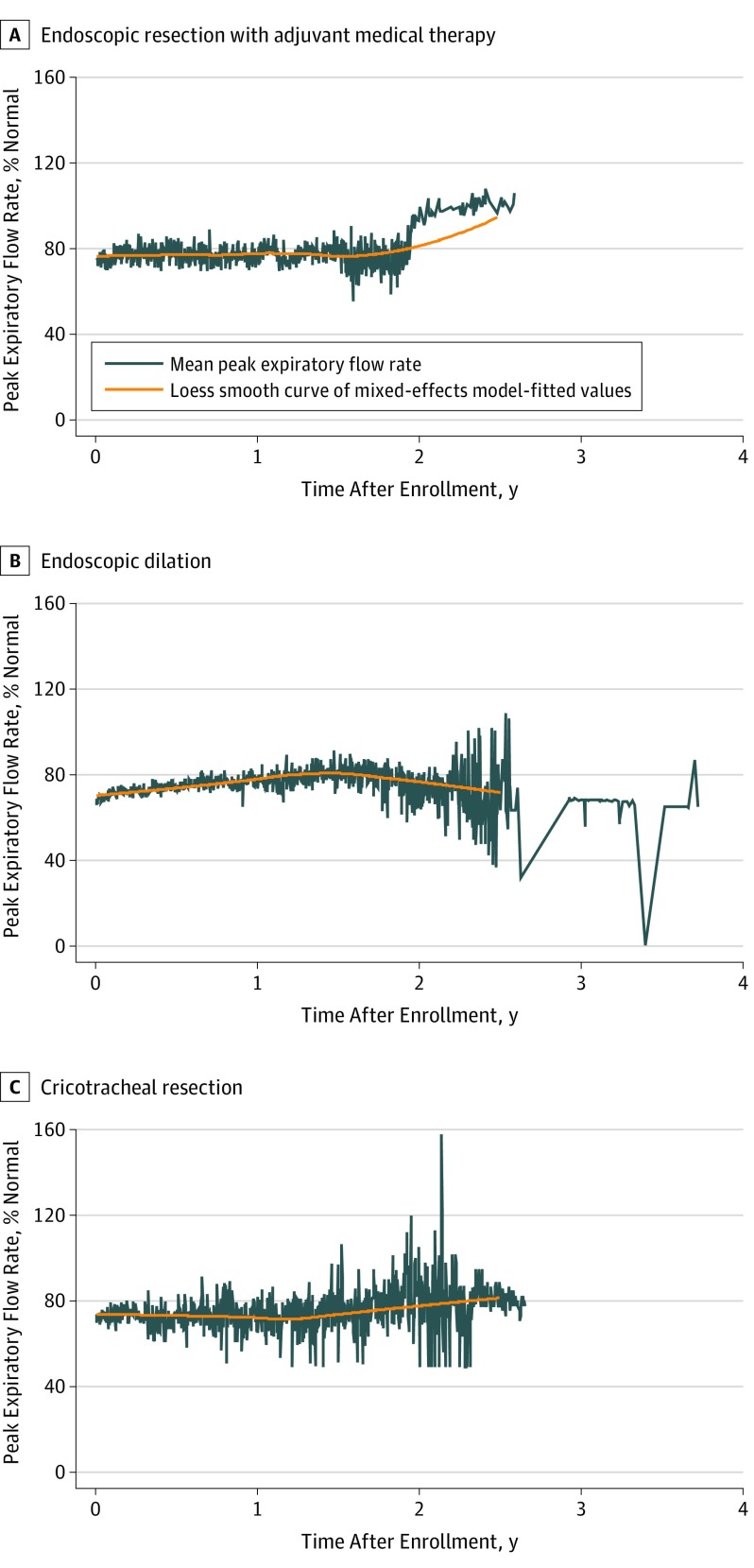
Longitudinal Mean Peak Expiratory Flow Rate Among Patients Without Recurrence in the 3 Treatment Arms Loess smooth curve of mixed-effects model shows sustained peak expiratory flow rate (measured in liters per second during a single expiratory cycle and reported as percentage of matched normative data) among patients after successful treatment. Self-reported patient longitudinal peak expiratory flow rate was captured using an inexpensive portable handheld device and a free smartphone app created specifically for this study.

In all, 496 (61.2%) patients recorded PEFR (ED: 383 [63.5%]; ERMT: 62 [51.2%]; and CTR: 51 [59.3%]); the only difference between reporters and nonreporters was that nonreporters were older at 52 years (95% CI, 43-59 years) vs 49 years (95% CI, 42-56 years). No difference in median %PEFR or CCQ score in the ED and ERMT groups was observed before recurrent surgical procedure (%PEFR: ED, 56.5% [IQR, 44%-69%] vs ERMT, 54% [IQR, 45%-59%] and CCQ: ED, 2.4 [IQR 1.6-3.4] vs ERMT, 2.6 [IQR, 1.6-3.3]). Thus, a similar threshold for repeat surgical procedure was exercised across modalities. Loess curves of mixed-effects model–fitted values for %PEFR confirmed that successfully treated patients without recurrence had stable respiratory function (Figure 3).

### Patient-Reported Outcome Measures

#### Breathing

**Figure 4.**
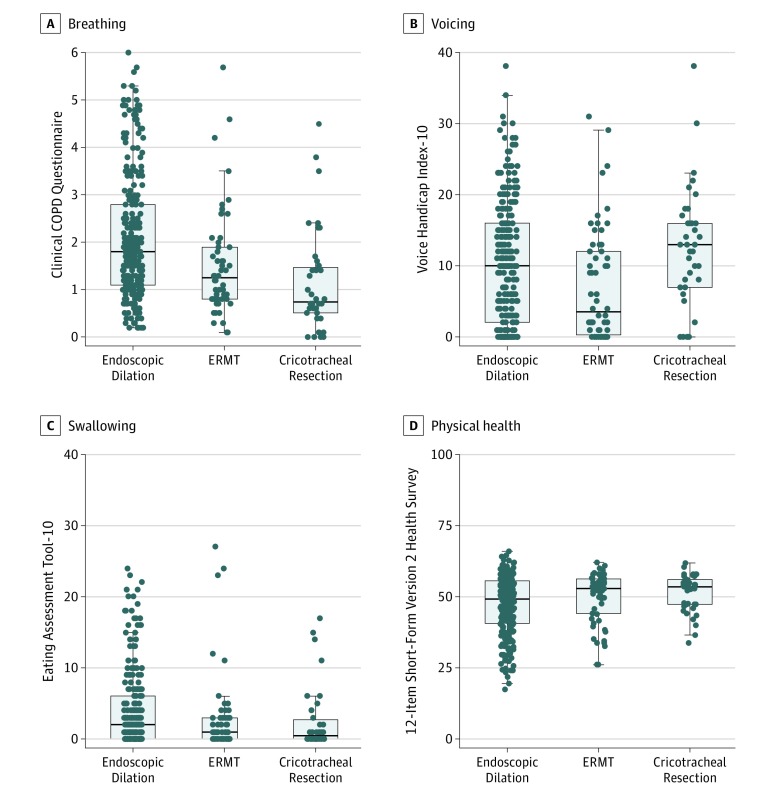
Secondary End Points of Patient-Reported Functional Outcome at 12 Months COPD indicates chronic obstructive pulmonary disease; ERMT, endoscopic resection with adjuvant medical therapy.

Among patients who were successfully treated (ie, did not recur during the study period), those who underwent CTR had the best CCQ scores at 360 days after enrollment followed by those who underwent ERMT and ED treatment (ED, 1.80 vs ERMT, 1.25 vs CTR, 0.75). Treatment with CTR was associated with statistically and clinically better breathing at 360 days compared with either ERMT or ED (CTR vs ERMT: ES, 0.3; 95% CI, 0-0.8; CTR vs ED: ES, 0.8; 95% CI, 0.4-1.3). Treatment with ERMT showed better breathing outcomes compared with treatment using ED (ERMT vs ED: ES, 0.5; 95% CI, 0.1-0.8) (Figure 4A).

#### Voice and Swallowing

Outcomes among successfully treated patients differed 360 days after enrollment. In contrast with breathing, Voice Handicap Index-10 scores of patients who underwent CTR were both statistically and clinically worse compared with scores for patients who underwent ERMT (CTR, 13.0 vs ERMT, 3.5; ES, 6; 95% CI, 1.0-10.0) but not different compared with ED (CTR, 13.0 vs ED, 10.0; *P* = .07). Patients who underwent ED had worse scores compared with those who underwent ERMT (ED, 10.0 vs ERMT, 3.5; ES, 3; 95% CI, 0-6.0) (Figure 4B). The association with swallowing was minimal among all treatment groups, and although statistical differences were observed, no Eating Assessment Test-10 scores met the threshold to be considered clinically abnormal (all median scores <3)^20^ (Figure 4C).

#### Global Physical Health

At 360 days, patients who underwent ED had lower median 12-Item Short-Form Version 2 scores compared with patients who underwent ERMT (ED, 49 vs ERMT, 53; ES, 2.3; 95% CI, −5.66 to 0.55) or CTR (ED, 49 vs CTR, 54; ES, 3.1; 95% CI, −6.9 to 0.29). These outcomes may suggest that global quality of life is statistically and clinically worse after treatment with ED compared with treatment of either ERMT or CTR (Figure 4D).

### Perioperative Complications and Death

Cricotracheal resection was associated with a number of complications. In order of incidence, 8 of 86 patients (9.3%) required a temporary tracheostomy or T-tube and 8 (9.3%) required an unplanned return to the operating room during their initial hospitalization. Moreover, 7 patients (8.1%) developed a permanent unilateral vocal fold paralysis, and 3 (3.5%) had an anastomotic leak. Less common complications included permanent bilateral vocal fold paralysis in 1 patient (1.2%), urinary tract infections secondary to an indwelling catheter in 1 patient (1.2%), and postoperative pneumonia in 1 patient (1.2%).

Endoscopic resection with adjuvant medical therapy was associated with adverse events during the surgical procedure and the postoperative medical regimen. In order of incidence, perioperative issues included 14 patients (11.6%) who developed temporary (≤4 weeks) tongue paresthesia associated with direct laryngoscopy and 4 patients (3.3%) who had a dental injury. Overall, 35 patients (28.9%) had an adverse reaction to trimethoprim/sulfamethoxazole received postoperatively, with 27 (22.3%) stopping the medication because of rash, nausea, or fever.

Endoscopic dilation had a similar perioperative adverse event profile compared with ERMT. These similarities included 84 of 603 patients (13.9%) who had temporary tongue paresthesia, 34 (5.6%) who had a dental injury, and 3 (0.5%) who had transient postoperative subcutaneous emphysema. All occurrences of subcutaneous emphysema resolved with conservative management. Of 810 patients, 3 died (0.04%): 1 in the ERMT group and 2 in the ED group. Death was secondary to airway obstruction more than 30 days after the surgical procedure.

## Discussion

Idiopathic subglottic stenosis is a recurrent, rare (1:400,000)^7^ fibroinflammatory disease that results in life-threatening blockage of the upper airway. Harnessing an engaged online community of patients coupled with innovative digital tools, we rapidly recruited a cohort of 810 patients with iSGS to compare the effectiveness of contemporary treatments with recurrent surgical procedure while assessing quality of life and perioperative risk trade-offs.

Treatment effectiveness at recurrent surgical procedures and risk trade-offs differed by modality. Patients who underwent CTR had the lowest rate of recurrent surgical procedure (1.2%), followed by ERMT (12.4%) and ED (28.0%). Considering disease recurrence in the context of treatment trade-offs, CTR was associated with the greatest perioperative risk and the worst postoperative patient-reported voice outcomes. Regarding disease recurrence rate, ERMT was at a 15.6% lower rate compared with ED (the present standard-of-care treatment). Both endoscopic procedures (ERMT and ED) had a similar low rate of perioperative risks and modest differences in patient-reported breathing, voice, and quality-of-life score changes compared with CTR. Swallowing complaints were uncommon regardless of treatment modality.

### Treatment Advantages and Trade-offs

#### Cricotracheal Resection

Our results are consistent with published single-center series documenting excellent surgical procedure outcomes after CTR.^26^ The disease recurrence rate after ERMT also parallels a retrospective analysis.^11^ Interestingly, published case series reported that 80% of patients had recurrent stenosis after undergoing ED within 1000 days of the initial surgical procedure.^11^ This result is a significantly higher rate than we observed in our study (28.0%). This disparity may stem from our inclusion of patients from centers of excellence and from smaller centers recruited online. Previous retrospective studies reported outcomes from high-volume centers with patients who have recalcitrant disease cluster, which may explain a higher disease recurrence rate.

Although our results appear to support the effectiveness and durability of CTR, they must be interpreted in context. First, not all patients were candidates for CTR because a sufficient distance below the vocal folds was required to perform this surgical procedure. Second, previous reports identified a significant rate of recurrent disease (10%-30%) among patients who underwent CTR that occurred from 5 to 10 years after the surgical procedure.^26,27,28^ These outcomes were not observed in our cohort given the temporal scope of our study. Longer-term follow-up of patients who underwent CTR from our cohort will continue to address this question. Although reports of patient complications after CTR are variable within the literature, our data appeared comparable with the reported 10% to 20% rate of anastomotic complications^26,27,28,29^ and 5% to 10% rate of postoperative unilateral vocal fold paralysis.^26,27^

#### Endoscopic Treatments

A notable outcome of our study was the findings for ERMT. In this 3-year study, ERMT offered significantly improved disease control compared with ED (the most common treatment) with minimal association with voice function, particularly when compared with CTR. Whether the reduced disease recurrence rate for ERMT was associated with surgical technique, postoperative medications, or a combination remains unclear. Moreover, whether ERMT outcomes will converge with ED during a longer follow-up is an area of continued study in our cohort.

#### Effectiveness of Novel Recruitment Strategy

For studies of rare disease, recruiting enough participants is challenging. Among clinical trials, 10% of investigators fail to recruit a single patient, and fewer than 20% of trials finish on time because of poor recruitment.^30^ Social media recruitment provides the ability to target a relevant and engaged audience of patients and allows for direct communication with participants. Although the issue of data privacy surrounding social media is clearly a salient topic, our study obtained no data from social media (ie, no content from social media profiles was queried or collected). We simply leveraged an online community of patients housed in social media for recruitment and to sustain patient engagement. Our ability to more than double our enrollment goal (target of 300 patients and actual recruitment of 810 patients) was a testament to the effectiveness of including a social media recruitment strategy. In our study, we believe this protocol promoted a deep sense of patient ownership in the study process and dramatically limited attrition (n = 35; 3.3%).

### Limitations

This study is not without limitations. Because of the nonrandom treatment assignment, unmeasured confounding variables may have affected the surgical outcomes. Additionally, given the individualized nature of surgical therapy, issues of generalizability exist in our results. CTR requires special training, experience, and institutional infrastructure, which limits the generalizability of our findings. The degree to which outcomes in our cohort for CTR are transferable between centers requires further study. Despite its advantages, ERMT was performed at only 1 institution and involved several intraoperative techniques that differed substantially from how most surgeons endoscopically treat iSGS. In addition, the postoperative medical regimen was complex. These factors may affect the generalizability of the results.

## Conclusions

We leveraged an engaged patient community on social media and collected patient-generated health data to study the natural history and outcomes of a rare airway disease. Our approach allowed nuanced comparison of the effectiveness of surgical treatments for iSGS. The most popular approach (ED) was associated with higher rates of recurrence compared with alternative treatments. Cricotracheal resection offered the most durable results but showed the greatest perioperative risk and worst long-term voice outcomes. Endoscopic resection with adjuvant medical therapy was associated with better disease control compared with ED, with minimal association with voicing. These results may be used to inform individual patient treatment decision-making and show the feasibility and effectiveness of integrating social media–based recruitment and patient-generated health data to drive the study of iSGS and other rare diseases.

## Notes

Supplement. **eFigure.** Study Patient Flow **eMethods.** Disease Specific Data, Multiple Imputation, and Propensity Score Matching **eTable 1.** Description of Propensity Score Matching Model Covariates in the Comparison (ED) and Treated (ERMT) Groups **eTable 2.** Propensity Score Matching: Cox Proportional Hazards Model **eReferences.**
