# Supplementary material for: Comparative Treatment Outcomes for Patients With Idiopathic Subglottic Stenosis
Source: JAMA Otolaryngol Head Neck Surg. 2019 Oct 31;146(1):20–9. doi: 10.1001/jamaoto.2019.3022 (PMC6824232; doi:10.1001/jamaoto.2019.3022)
Supplement: Supplement. — eFigure. Study Patient Flow eMethods. Disease Specific Data, Multiple Imputation, and Propensity Score Matching eTable 1. Description of Propensity Score Matching Model Covariates in the Comparison (ED) and Treated (ERMT) Groups eTable 2. Propensity Score Matching: Cox Proportional Hazards Model eReferences. [file jamaotolaryngolheadnecksurg-146-20-s001.pdf]

Supplementary Online Content

Gelbard A, Anderson C, Berry LD, et al. Comparative treatment outcomes for patients with idiopathic subglottic stenosis. *JAMA Otolaryngol Head Neck Surg*. Published online October 31, 2019. doi:10.1001/jamaoto.2019.3022

eFigure. Study Patient Flow

eMethods. Disease Specific Data, Multiple Imputation, and Propensity Score Matching

eTable 1. Description of Propensity Score Matching Model Covariates in the Comparison (ED) and Treated (ERMT) Groups

eTable 2. Propensity Score Matching: Cox Proportional Hazards Model

eReferences.

This supplementary material has been provided by the authors to give readers additional information about their work.

© 2019 Gelbard A et al. *JAMA Otolaryngology-Head & Neck Surgery*.

eFigure. Study Patient Flow

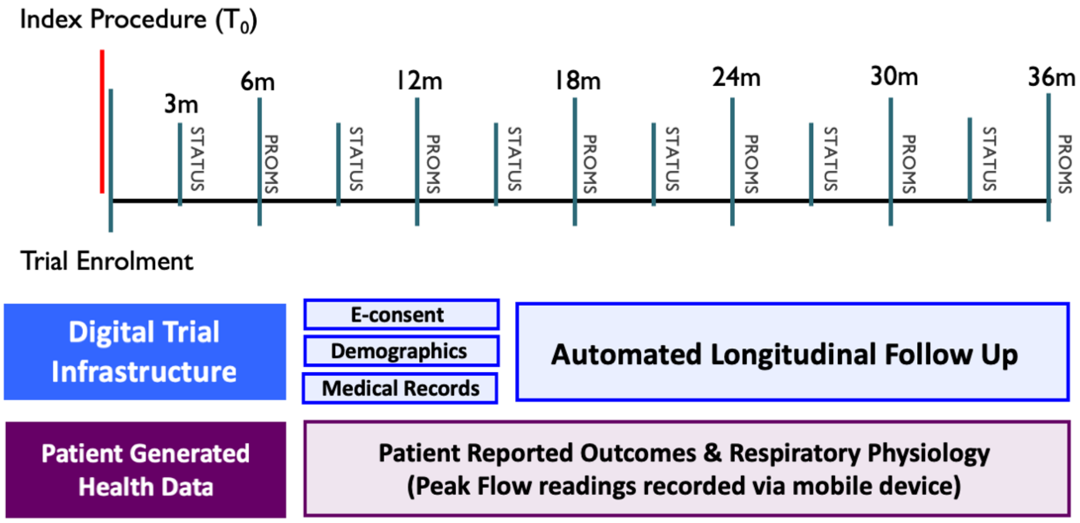

eFigure 1. Schematic overview of NoAAC PR02 trial workflow. Data collection was implemented in an electronic data capture (EDC) system recording clinical data along with patient generated health data (consisting of validated patient reported outcome measures, and longitudinal peak expiratory flow rate recorded into a smartphone app)

© 2019 Gelbard A et al. *JAMA Otolaryngology-Head & Neck Surgery*.

eMethods.

Disease-specific Data

Anatomic disease characteristics collected included surgeon-reported percent luminal compromise, distance of scar from vocal folds [mm], and craniocaudal extent of scar [mm]. A mucosal atopy index score was assigned to each patient (1 point for each of the following conditions representing allergic/atopic disease at a mucosal interface: allergic rhinitis, chronic sinusitis, asthma, eczema, Crohn's disease, ulcerative colitis. A composite index score was obtained by dividing the number of points by 6). Comorbid disease burden was determined based on the non-age-adjusted Charlson Comorbidity index (CCI)[4] and select criteria from European Laryngological Society consensus statement[5] (vocal cord mobility and presence of gastroesophageal reflux disease).

© 2019 Gelbard A et al. *JAMA Otolaryngology-Head & Neck Surgery*.

Multiple Imputation

Multiple imputation was used to address missing covariate values. With a survival outcome, the event indicator, the Nelson-Aalen estimator of the cumulative hazard rate, and covariates were included in the imputation model[6]. We repeated the imputation five times, independently. The imputed datasets were analyzed separately using Cox proportional hazards models. parameter estimates were averaged over five sets of analysis results. The variance estimators of the averaged parameters were estimated using Rubin's multiple imputation[7].

Propensity Score Matching

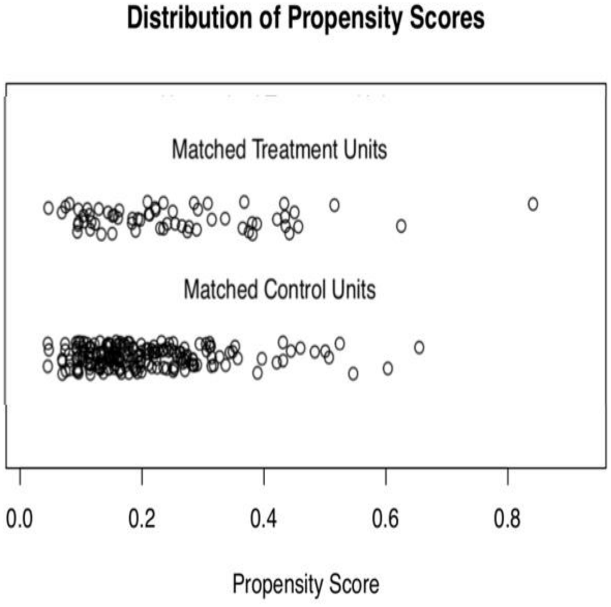

Figure S2. Verification of common support in Propensity Score Matching demonstrates evenly matched scores in Treated (ERMT) and Controls (ED).

Propensity score matching (PSM) addressed the effect of observed confounders. iSGS is a rare disease with a limited evidence base. Thus, we created propensity score–matched cohorts of ED and ERMT patients using covariates determined a priori by expert consensus on the hypothesized effect of the variable on the type of surgical treatment or the outcome. Logistic regression generated the propensity score, defined as the probability of receiving ED, conditional on covariates. Covariates included in the model were age, highest education level, marital status, non-age-adjusted Charlson comorbidity index, Mucosal Atopy index, GERD clinical diagnosis, number of prior surgeries, estrogen exposure, and age at first parturition. Nearest neighbor matching (NNM) without replacement was used to select the patients in the ED cohort to include in the final data set. No caliper was used. After constructing the propensity score, ERMT subjects were matched 3:1 with ED subjects (**Figure S2**). Analyses were performed using R statistical software (R version 3.4.3) with package 'MatchIt'.

We selected covariates known to affect treatment selection. These primarily included sociodemographic characteristics (age, highest education level, and marital status). These variables have demonstrated associations with the ability to travel large distances to specific medical centers, or to affect how severe a patient's disease was at presentation Additionally, we included variables believed to be related to the outcome but not necessarily the treatment to reduce bias.[8] Comorbidity burden captured by the Charlson comorbidity index score, as well as specific comorbid conditions

© 2019 Gelbard A et al. *JAMA Otolaryngology-Head & Neck Surgery.*

(captured in the binary assessment of gastroesophageal disease, and the score of the airway mucosal atopy index) were hypothesized to effect either treatment allocation or outcomes. Similarly, estrogen exposure (time from menarche to menopause adjusted for use of hormone replacement therapy, and number of pregnancies), and disease severity (number of prior procedures) were hypothesized to affect both treatment allocation and outcomes. Once the propensity score was calculated, overlap was ensured in the range of propensity scores across treatment and comparison groups (i.e., verified "common support") by examining a graph of propensity scores across treatment and comparison groups **(Figure S2**). Similar distribution (balance) was assessed by splitting the sample by quintiles, and then verifying an equivalent mean propensity score in the treatment and comparison groups within each quintile.[9] Unbalanced quintiles were split into smaller blocks, and balance was achieved (**Figure S3**). We verified successful matching by estimating the effect of treatment on disease outcomes (**Table S1**). Proposed maximum standardized differences for specific covariates range from 10 to 25 percent.[10,11]

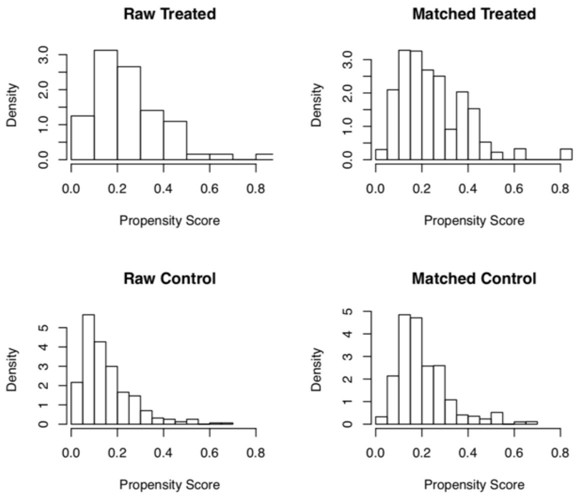

Figure S3. Verification of similar distribution or "balance" in the treated (ERMT) and comparison (ED) groups. Distribution estimated by splitting the sample by quintiles, followed by verification of equivalence of mean propensity score in the treatment and comparison groups within each of the five quintiles. Unbalanced quintiles were split into smaller blocks, where balance was achieved

eTable 1. Description of Propensity Score Matching Model Covariates in the Comparison (ED) and Treated (ERMT) Groups

Table S1.	ERMT *N* = 52		ED *N* = 307		*P*
Age (at index)	52 (47- 58)		48 (40-56)		*P=0.42*
Marital Status					*P=0.87*
Married	88%	*(46)*	88%	*(269)*	
non-Married	12%	*(6)*	12%	*(38)*	
Highest education level					*P=0.93*
Graduate school	19%	*(10)*	20%	*(60)*	
College	46%	*(24)*	50%	*(155)*	
Some college	25%	*(13)*	23%	*(70)*	
High School	10%	*(5)*	7%	*(22)*	
Charlson Comorbidity Index					*P=0.88*
0	90%	*(47)*	91%	*(278)*	
1	8%	*(4)*	6%	*(18)*	
2	2%	*(1)*	3%	*(10)*	
3	0%	*0*	0%	*(1)*	
Mucosal Atopy Index					*P=0.18*
0	63%	*(33)*	43%	*(132)*	
0.17	27%	*(14)*	33%	*(101)*	
0.33	4%	*(2)*	16%	*(49)*	
0.5	6%	*(3)*	7%	*(21)*	
0.67	0%	*0*	1%	*(4)*	
Estrogen Exposure (years)	26 (22- 34)		27 (19-32)		*P=0.21*
Full Term Pregnancy					*P=0.1*
0	8%	*(4)*	20%	*(60)*	
1	4%	*(2)*	14%	*(43)*	
2	48%	*(25)*	43%	*(133)*	
3	25%	*(13)*	14%	*(42)*	
More than 3	15%	*(8)*	9%	*(29)*	
Number of prior surgeries	3.5 (2.0- 6.5)		3.0 (2.0-8.0)		*P=0.075*

Table S1. Description of PSM model covariates in the comparison (ED) and treated (ERMT) groups. Successful matching of the covariates in our model indicated by lack of statistical differences between covariates in each group. ED: Endoscopic Dilation, ERMT: Endoscopic Resection with Adjuvant Medical Therapy.

When employing PSM to compare the probability of recurrent procedure between the ED and ERMT groups, we saw consistent results with our prior standard models. ED had a hazard ratio of 2.77 (IQR: 1.4–5.5), and this effect persisted in PSM models employing multiple imputation: ED vs. ERMT HR: 3.16 (IQR: 1.8 – 5.5) (**Table S2**)

© 2019 Gelbard A et al. *JAMA Otolaryngology-Head & Neck Surgery*.

eTable 2. Propensity Score Matching: Cox Proportional Hazards Model

eTable 2. Propensity Score Matching: Cox Proportional Hazards Model

Hazard Ratio for Recurrence: Propensity Scoring Matching (without multiple imputation)	Hazard Ratio	Lower 95%	Upper 95%
Procedure Type - ED : ERMT	2.7799	1.402	5.5123

Hazard Ratio for Recurrence: Propensity Scoring Matching (with multiple imputation)	Hazard Ratio	Lower 95%	Upper 95%
Procedure Type - ED : ERMT	3.1646	1.8188	5.5062

Table S2. Propensity score matched models comparing rate of disease recurrence after surgical treatment*. ED was inferior to ERMT (HR 2.77, 95% CI 1.4 – 5.5), and this effect persisted in PSM models accounting for missing data (ED vs. ERMT, HR 3.16, 95% CI 1.8 – 5.5). **Since there was only one recurrent procedure in the CTR group, we did not compare adjusted recurrent procedure rates between CTR and the other two groups.*

© 2019 Gelbard A et al. *JAMA Otolaryngology-Head & Neck Surgery*.

eReferences

1. Vanderbilt. Airflo Mobile Health Data Monitoring Tool. 2019; https://itunes.apple.com/us/app/airflo/id1032827896?mt=8.
2. Hankinson JL, Odencrantz JR, Fedan KB. Spirometric reference values from a sample of the general U.S. population. *Am J Respir Crit Care Med.* 1999;159(1):179-187.
3. Carpenter DJ, Ferrante S, Bakos SR, Clary MS, Gelbard AH, Daniero JJ. Utility of Routine Spirometry Measures for Surveillance of Idiopathic Subglottic Stenosis. *JAMA otolaryngology-- head & neck surgery.* 2018.
4. Charlson ME, Pompei P, Ales KL, MacKenzie CR. A new method of classifying prognostic comorbidity in longitudinal studies: development and validation. *J Chronic Dis.* 1987;40(5):373-383.
5. Monnier P, Dikkers FG, Eckel H, et al. Preoperative assessment and classification of benign laryngotracheal stenosis: a consensus paper of the European Laryngological Society. *Eur Arch Otorhinolaryngol.* 2015;272(10):2885-2896.
6. Sterne JA, White IR, Carlin JB, et al. Multiple imputation for missing data in epidemiological and clinical research: potential and pitfalls. *BMJ.* 2009;338:b2393.
7. Hughes RA, Sterne J, Tilling K. Comparison of imputation variance estimators. *Stat Methods Med Res.* 2016;25(6):2541-2557.
8. Brookhart MA, Schneeweiss S, Rothman KJ, Glynn RJ, Avorn J, Sturmer T. Variable selection for propensity score models. *Am J Epidemiol.* 2006;163(12):1149-1156.
9. GW I. Nonparametric Estimation of Average Treatment Effects under Exogeneity: A Review. *Review of Economics and Statistics.* 2004;86(1):4-29.
10. Stuart EA, Lee BK, Leacy FP. Prognostic score-based balance measures can be a useful diagnostic for propensity score methods in comparative effectiveness research. *Journal of clinical epidemiology.* 2013;66(8 Suppl):S84-S90 e81.
11. Austin PC. Balance diagnostics for comparing the distribution of baseline covariates between treatment groups in propensity-score matched samples. *Statistics in medicine.* 2009;28(25):3083-3107.

© 2019 Gelbard A et al. *JAMA Otolaryngology-Head & Neck Surgery.*
